# Local and Metastatic Relapse Features in Patients After a Primary Soft Tissue Sarcoma: Advocating for a Better-Tailored Follow-Up

**DOI:** 10.3389/fonc.2019.00559

**Published:** 2019-07-02

**Authors:** Céline Blaye, Michele Kind, Eberhard Stoeckle, Véronique Brouste, Guy Kantor, François Le Loarer, Antoine Italiano, Maud Toulmonde

**Affiliations:** ^1^Department of Medical Oncology, Institut Bergonié, Bordeaux, France; ^2^Department of Radiology, Institut Bergonié, Bordeaux, France; ^3^Department of Surgical Oncology, Institut Bergonié, Bordeaux, France; ^4^Department of Clinical and Epidemiological Research, Institut Bergonié, Bordeaux, France; ^5^Department of Radiation Oncology, Institut Bergonié, Bordeaux, France; ^6^Department of Pathology, Institut Bergonié, Bordeaux, France

**Keywords:** sarcoma, retrospective study, follow-up, relapse, guidelines

## Abstract

**Background:** No consensus exists on how to follow patients after complete remission of a primary Soft Tissue Sarcoma (STS). Studying relapse features could help tailor guidelines for follow-up.

**Patients and Methods:** Patients in complete remission after initial management of a localized STS at Institut Bergonié who presented a first local and/or metastatic relapse between January 1995 and July 2015 were eligible. Characteristics of relapse diagnosis were retrospectively collected.

**Results:** 359 patients met inclusion criteria. 197 and 187 patients presented a local relapse and a metastatic relapse, respectively. In group 1 (limbs/trunk wall) and 2 (trunk/gynecological/other location), local relapse was diagnosed on clinical symptoms in 89 and 44% of cases, first detected by the patient himself in 68.5 and 34% of cases, and outside a planned visit in 67 and 36% of cases, respectively. In patients with metastatic relapse, diagnosis was made during a planned visit in 63% of cases, and by imaging in 62% of cases. Median survival after relapse was not different whether the first local relapse was diagnosed clinically or by imaging (44 [95%CI: 28–69.8] vs. 57 months [95%CI: 33.9–84.5], *p* = 0.35) but was longer if diagnosis of metastatic relapse was made on planned chest-CT scan rather than chest X-ray (58 [95%CI: 35.5–103.9] vs. 25 months [95%CI: 16.5–32.6], *p* < 0.05).

**Conclusion:** Patient's education for regular clinical examination can be recommended for follow-up of local relapses after a primary STS of the limbs or superficial trunk. Modeling studies aiming at better understanding and predicting tumor biology to improve tailoring STS patients' follow-up are warranted.

## Introduction

Soft-tissue sarcomas (STS) are rare and very heterogeneous tumors (1, 2) with an estimated incidence averaging 4–5/100,000/year in Europe (3). Despite a better knowledge of relapse's risk factors (4), and advocating for management in specialized centers (5), nearly half of patients will experience relapse (3). Relapses mostly occur during the 5 years following initial treatment, and up to 80% of them happen during the first 2 years of follow-up (6). However, late recurrences—i.e., recurrences occurring more than 5 years after initial management—are not uncommon in specific groups of patients (7).

The hypothesis behind follow-up after a cancer is that early detection of relapse can lead to a better prognostic, such as a second chance of cure or at least a longer disease control. However, no consensus exists on how to follow patients after complete remission of a primary STS (4). Moreover, relapse characteristics in this heterogeneous disease are largely unknown.

We hypothesized that better characterization of relapse features could help tailor guidelines for follow-up. In this study, we aimed to describe relapse features in a cohort of patients homogeneously treated and followed at a single institution after complete remission of a localized primary STS.

## Materials and Methods

### Patients

We retrospectively searched our institutional database (conticabase.sarcomabcb.org) for patients meeting inclusion criteria as follow: (1) a primary localized STS (no lymph nodes nor metastasis at diagnosis); (2) treated by surgery (and chemotherapy and/or radiotherapy if indicated); (3) considered in complete remission at the end of initial management; (4) experiencing a first event of local (LR) and/or metastatic (MR) relapse; (5) diagnosed between January 1995, and July 2015. Gastrointestinal stromal tumors (GIST) were not included in the study.

### Methods

Timing and type of follow up were guided by ESMO guidelines (4) and can be found in Appendix.

Patient's medical charts were retrieved to collect patients, primary tumor and initial management characteristics as well as relapse diagnosis features and survival data (Appendix). Date of surgery was considered the date of complete remission.

The event of interest in this study was the first relapse. Following features were collected: (1) Type of relapse: local or metastatic. If both were present concomitantly, the location that led to the diagnosis was retained, but patients were included in the metastatic group for survival analysis, because of a worse prognosis (8); (2) Type of visit leading to relapse: planned or unplanned. To assess this modality, date and place of last visit before visit conducting to relapse diagnosis was also collected; (3) Mode of diagnosis: clinical examination or imaging. If the mode of diagnosis was clinical, clinical symptoms were collected; if the mode of diagnosis was imaging, the date and type of imaging leading to diagnosis was collected; (4) Origin of diagnosis: patient or physician.

Because timing and type of follow up for local relapse differ according to location, patients were further classified between the limbs/trunk wall group (tumors that were palpable at diagnosis) (group 1) and the internal trunk/gynecological/other location group (group 2).

### Statistical Analysis

Qualitative data were given in number and percent. Quantitative data were given with median. Overall Survival (OS) probability was estimated using the Kaplan-Meier method and compared with the log-rang test. The time of study participation was defined from tumor diagnostic to death. Every death (due to cancer or not) was considered as an event. Patients lost to follow-up or alive at the end of the analyses were censored. Statistical significance level was set at *p* < 0.05.

## Results

Between January 1995 and July 2015, 359 patients experienced a first local (LR) or metastatic (MR) relapse after complete remission of a primary localized STS treated at our institution, and were included in this study. Patient, primary tumor and treatment characteristics are detailed in Table 1.

**Table 1:** Patients' characteristics at initial management (*N* = 359) (group 1: limbs and trunk wall, group 2: internal trunk, gynecological, head, and neck).

		**All patients**		**Group 1**		**Group 2**	
		**(*****N*** **=** **359)**		**(*****N*** **=** **199)**		**(*****N*** **=** **160)**	
		***n***	**%**	***n***	**%**	***n***	**%**
Gender	Male	179	50	116	58	63	39
	Female	180	50	83	42	97	61
Age at diagnosis	Median	60		61		58	
	Min-max	14–88		14–88		21–86	
Location of primary tumor	Limbs	133	37	133	67		
	Trunk wall	66	18	66	33		
	Internal trunk	102	28.5			102	64
	Gynecological	41	11.5			41	25.5
	Head and neck	17	5			17	10.5
Size (mm)	Median	90		75		100	
	Min-max	5–500		10–500		5–450	
Depth	Deep	270	75	117	59	153	95.5
	Superficial	35	10	31	15.5	4	2.5
	Both	54	15	51	25.5	3	2
Histology	Leiomyosarcoma	98	27	37	19	61	38
	Liposarcoma	81	23	32	16	49	31
	UPS or NOS	52	14.5	38	19	14	9
	Others	128	35.5	92	46	36	22
Grade	3	171	48	108	54	63	39
	2	121	34	61	31	60	38
	1	31	8	14	7	17	11
	Unknown	36	10	16	8	20	12
Type of surgery	Amputation/wide resection	252	70	151	76	101	63
	Simple resection/enucleation	103	29	46	23	57	36
	Unknown	4	1	2	1	2	1
Resection margins	R1	158	44	80	40	78	49
	R0	145	40	106	53	39	24
	Unknown	56	16	13	7	43	27
Tumor spillage	Yes	42	12	17	9	25	16
	No	239	67	135	68	104	65
	Unknown	78	21	47	23	31	19
Radiotherapy	Yes	197	55	137	69	60	37.5
	No	162	45	62	31	100	62.5
Chemotherapy	Neo ± adjuvant	84	23	63	32	21	13
	Adjuvant	46	13	28	14	18	11
	No	229	64	108	54	121	76

### Local Relapses

One hundred ninety seven patients experienced local relapse. Of them, 25 patients had concomitant local and metastatic relapse, with local relapse leading to diagnosis. In more than half of the cases, LR was diagnosed out of planned follow-up (“interval” relapse: 104 patients, 53%). The person leading to the diagnosis of relapse was the patient in 106 cases (54%). Clinical findings led to diagnosis in 136 cases (69%), whereas imaging found 61 LR (31%). Most frequent symptoms associated with clinical diagnosis were swelling (96 patients, 71%) and pain (17 patients, 12.5%), or both (11 patients, 8%). When imaging led to diagnosis, it was mostly CT-scan (46 patients, 75%) and MRI (11 patients, 18%).

**Figure 1:**
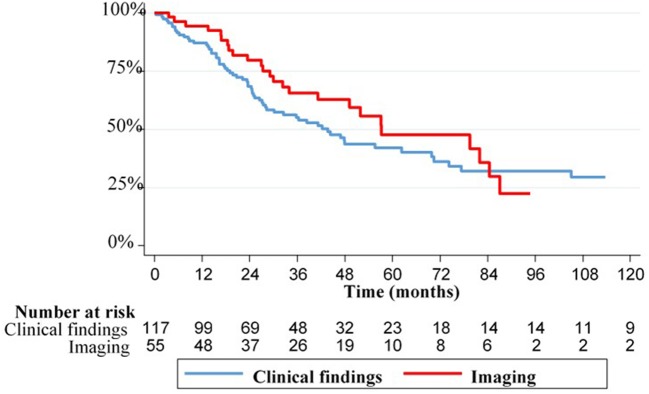
Kaplan-Meier curves of overall survival for patients with local relapse according to relapse features.

Median survival for patients presenting with LR only was 48 months (95%CI: 36.3–69.8). Median survival was 41 months (95%CI: 25.0–74.2) for patients diagnosed with interval LR vs. 57 months (95%CI: 41.3–82.0) for patients diagnosed with LR on planned follow-up (*p* = 0.5). Median survival was 44 months (95%CI: 28.0–69.8) for patients with LR diagnosed on clinical findings vs. 57 months (95%CI: 33.9–84.5) for patients with LR diagnosed on imaging (*p* = 0.35) (Figure 1).

#### In the Group 1 (Limbs and Trunk Wall Primary Location, n = 199)

Ninety-two patients experienced local relapse, and 10 patients had concomitant local and metastatic relapse, with local relapse leading to diagnosis. In patients with LR only, 62 (67%) had interval LR. The person leading to the diagnosis of LR was the patient in 63 cases (68.5%). Clinical findings led to diagnosis in 82 cases (89%), whereas imaging diagnosed 10 LR (11%). Symptoms associated with clinical diagnosis were swelling (67 patients, 82%) and pain (8 patients, 10%), or both (5 patients, 6%). Imaging leading to diagnosis was mostly MRI (7 patients, 70%).

Median survival for patients presenting with LR only in group 1 was 57 months (95%CI: 41.2–77.3). Median survival was 70.5 months (95%CI: 55.6–146.6) for patients with LR diagnosed on planned follow-up vs. 42 months (95%CI: 25.3–77.3) with interval LR (*p* = 0.15). Median survival was 146.6 months (95%CI: 16.8–146.6) for the 10 patients with LR diagnosed on imaging vs. 47.8 months (95%CI: 35.7–74.2) for the 82 patients with LR diagnosed on clinical findings (*p* = 0.13).

#### In the Group 2 (Internal Trunk, Gynecological, and Head and Neck Primary Location n = 160)

Eighty patients experienced local relapse, and 15 patients had concomitant local and metastatic relapse, with local relapse leading to diagnosis. In patients with LR only, 29 (36%) were diagnosed with “interval” relapse. The person leading to the diagnosis of LR was the patient in 27 cases (34%). Clinical findings led to diagnosis in 35 cases (44%), whereas imaging diagnosed 45 LR (56%). Most frequent symptoms associated with clinical diagnosis were swelling (51%) and pain (17%), or both (11%). Imaging leading to diagnosis was mostly CT-scan (40 patients, 89%).

Median survival for patients presenting with LR only in group 2 was 41 months (95%CI: 27.4 −79.5). Median survival was 44 months (95%CI: 29.1–79.5) for patients with LR diagnosed on planned follow-up vs. 25 months (95%CI: 20.7–NR) with interval LR (*p* = 0.9). Median survival was 52 months (95%CI: 30.0–82.0) for patients with LR diagnosed on imaging vs. 25 months (95%CI: 20.7–NR) for patients with LR diagnosed on clinical findings (*p* = 0.23).

We performed an exploratory analysis on LR features in the subgroup of the 102 patients with initial internal trunk sarcoma. Sixty presented a LR, and 6 had a LR associated with metastasis. Relapse was diagnosed during follow-up for 47 patients (71%), by a physician for 47 patients (71%), and on imaging for 41 patients (62%). The most common imaging was CT scan in 40 cases (39%). The median survival was 52 months (95%CI: 29.1–82) for patients with LR diagnosed on planned follow-up vs. 23 months (95%CI: 5.3–24.6) for interval LR (*p* = 0.02). Median survival was 52 months (95%CI: 29.1–84.5) for patients with LR diagnosed on imaging, vs. 23.5 months (95%CI: 13.8–27.4) for patients with LR diagnosed on clinical findings (*p* = 0.02).

### Metastatic Relapses

One hundred eighty-seven patients experienced metastatic relapse. Of them, 30 had concomitant local and metastatic relapse: five with metastatic relapse first leading to diagnosis, and 25 with local relapse first leading to diagnosis. One hundred fifty eight patients (84.5%) had only one site of metastasis, and 117 patients (63%) had pulmonary metastasis. Other metastatic locations were bones (15 patients, 8%), peritoneum (13 patients, 7%), and liver (12 patients, 6%).

Diagnosis was made during planned follow-up in 102 patients (63%), on imaging in 101 patients (62%), and on clinical symptoms in 61 patients (38%). The person leading to the diagnosis of relapse was more often a physician (109 patients, 67%). The most frequent symptoms were pain (22 patients, 36%), swelling (16 patients, 26%), and respiratory symptoms (11 patients, 18%). Imaging for diagnosis of MR included chest X-ray (49 patients, 48.5%) and CT-scan (46 patients, 45.5%).

Median survival after MR was 27 months (95%CI: 21.6–35). Patients with interval MR had a median survival of 21 months (95%CI: 13.2–27.0) vs. 33 months (95%CI: 25.0–40.6) for patients with diagnostic of MR on planned follow up (*p* = 0.02). Median survival was 21 months (95%CI: 12.5–26.9) if diagnosis was made on clinical symptoms vs. 35 months (95%CI: 25.0–40.6) if diagnostic was made on imaging (*p* < 0.01).

For patients diagnosed with pulmonary MR during planned follow-up, median survival after relapse was 58 months (95%CI: 35.5–103.9) if diagnosed on CT-scan vs. 25 months (95%CI: 16.5–32.6) if diagnosed on chest X-ray (*p* < 0.01). In order to assess whether the difference in survival could be linked to an artificial earlier diagnosis with CT-scan, we assessed survival curves from disease diagnosis. Overall survival analysis from the diagnosis showed a median survival of 102 months (95%CI: 63.7–not reached) for patients diagnosed with CT-scan, vs. 52 months for patients diagnosed with chest X-ray (95%CI: 37.5–64.0) (*p* < 0.01), whereas median time between initial diagnosis and metastatic relapse diagnostic was 36 months in both groups.

## Discussion

This study reports features of relapse diagnosis in a population of patients treated for a primary localized STS in a comprehensive manner in a center where clinical follow-up was predominant.

In this study, the large majority of local relapse diagnoses were made by patients themselves in-between planned visits. In the group of patients with limb/trunk wall sarcoma, there was no significant difference in survival between patients with diagnosis of local relapse made on imaging vs. clinically. These results suggest that clinical assessment can be sufficient for efficient follow-up of local relapse for patients in remission of a limb/trunk wall sarcoma. This also emphasizes the important role of patient's education in this setting. These results are in concordance with previous studies showing that systematic MRI for detecting local relapse is not needed for limb and trunk wall sarcoma follow-up. Imaging should be reserved for primary tumor sites that are not readily assessed by history and physical examination, as advised by the NCCN recommendations (9, 10).

The survival of our patients with internal trunk primary tumor was better when relapse was diagnosed on imaging than clinical findings. Indeed, patients with localized relapse of retroperitoneal sarcoma can be candidates to iterative surgeries with subsequent long complete remission periods (11, 12). Unfortunately, we did not assess the percentage of patients with subsequent surgeries for local relapse and their outcome. In group 1, recurrences occurred with a median of 22 months and could occur up to 18 years later. It seems therefore important to maintain regular and long-term imaging follow-up for these patients specifically. However, optimal timing and length are currently unknown. Joensuu et al. published an interesting work on optimizing timing of CT scans in the follow-up of patients treated for a GIST based on their biological risk of relapse. They showed that detection of GIST recurrence may be enhanced by adjusting the timing of the CT scans with the hazard of recurrence in time (13). Performing such study in patients with internal STS according to tumor biology factors (i.e., histology, grade, and mitotic count) could help better tailor imaging follow-up in this specific group.

Half of our patients eventually presented with metastasis. The median survival after metastatic relapse was 27 months. This was longer when compared to previous recent studies in this setting (14). Importantly, one third of patients had a diagnosis of metastatic relapse made outside regular imaging follow-up. These patients had poor survival, as well as those with clinical diagnosis of metastatic relapse rather than on imaging. This demonstrates the aggressive evolutionary kinetic of interval disease, and ultimately the leading role of tumor biology in prognosis. Altogether, this data clearly questions the limits of regular follow-up in these cases (15). These results also support the growing interest in developing predictive models of tumor growth patterns based on biological parameters that could allow tailoring STS patients follow-up for metastatic relapse on a more individual level (16).

We report a better survival for patients whose diagnosis of lung metastasis was made on CT-scan rather than X-ray. This could be explained by several reasons. The first is an artificial earlier diagnosis of metastases on CT-scan conferring a false improvement in survival (17). In our study, however, the lung metastases detection did not seem to happen earlier on CT-scan than X-ray. The second explanation is a selection bias in this retrospective cohort. Indeed, 49% of patients diagnosed on chest X-ray were diagnosed between 1994 and 2005 vs. 23% on CT-scan. This could at least partly explain the difference observed in this study, since survival of metastatic patients has improved among years (14). Unfortunately, only one small prospective trial published to date assessed chest X-ray vs. CT-scan in the follow-up of STS patients. This was a secondary endpoint and the study could not demonstrate a difference in disease free survival between the two types of imaging (24). The third possible reason is that CT scan earlier detection of metastatic disease could allow local treatment of smaller metastasis that could confer a better survival to carefully selected patients (18–22). Unfortunately we could not assess this point in this retrospective study. Interestingly, a recent national study conducted by the French Sarcoma Group has demonstrated that loco-regional treatment of metastatic disease was an independent prognostic factor of better survival in the metastatic setting (23). The optimal way to select patients who could benefit from this approach–and could therefore be the best candidates for regular imaging follow-up–still needs to be determined.

## Conclusion

In patients in complete remission after a primary STS of the limbs or trunk wall, patient's education for regular clinical examination can be recommended for follow up of local relapse, whereas in patients with internal STS non-clinically assessable at diagnosis, imaging is the main modality of follow-up. However, timing of this imaging still needs to be optimized. Most metastatic relapses are detected on imaging but one third can happen out of planned follow-up due to symptoms, and this is associated with poorer survival. This demonstrates the superiority of tumor biology in disease course and the limits of imaging follow-up in patients with very aggressive disease. With this in mind, the unbiased impact and interest of follow-up with chest CT scan compared to chest X-ray on patients' management and survival remains to be determined. Overall, modeling studies aiming at better understanding and predicting tumor biology to improve tailoring patients' follow-up after a STS are warranted.

## Ethics Statement

This study was approved by the IRB of Institut Bergonié.

## Author Contributions

All authors listed have made a substantial, direct and intellectual contribution to the work, and approved it for publication.

### Conflict of Interest Statement

The authors declare that the research was conducted in the absence of any commercial or financial relationships that could be construed as a potential conflict of interest.

## Abbreviations

LR: local relapse
MR: metastatic relapse
STS: soft tissue sarcoma.
