# Supplementary material for: Local and Metastatic Relapse Features in Patients After a Primary Soft Tissue Sarcoma: Advocating for a Better-Tailored Follow-Up
Source: Front Oncol. 2019 Jul 2;9:559. doi: 10.3389/fonc.2019.00559 (PMC6614176; doi:10.3389/fonc.2019.00559)
Supplement: Supplementary file 1 [file Table_1.docx]

Appendix 1

MATERIAL and METHODS

Modalities of follow up

Globally, for limbs and trunk wall location, patients were followed with clinical examination and chest X-ray every 3 to 4 months during the first three years if considered high risk (high grade tumors, deep primary location, tumor size > 5 cm) or every 6 months if considered low risk; then twice a year during two years, and generally every year for up to ten years if high risk.

High risk patients with uneasily palpable lesion would also have an annual MRI.

For other deep locations such as internal trunk, clinical examination could be performed every 3 to 6 months and CT-scan every 6 months during 2 to 3 years, and generally once a year for up to ten years. Follow-up was sometimes alternating with the general practitioner after the 2 first years of follow-up, and could be continued entirely by the general practitioner after 5 years.

Characteristics collected

Patients’ characteristics included birth date, age at diagnosis and gender.

Primary tumor characteristics included location, size, depth, histology, grade and differentiation. The histological diagnosis was made according to the World Health Organization Classification of Tumors; histological grade was determined according to the Fédération Nationale des Centres de Lutte Contre le Cancer (FNCLCC) grading system.

Initial management characteristics included date of surgery, type of surgery (wide or sparing), resection margins (R0 or R1), tumor spillage, perioperative radiotherapy and/or chemotherapy.
